# Clinical and transcriptional recovery profiles in pediatric and adult multiple sclerosis patients

**DOI:** 10.1002/acn3.51244

**Published:** 2020-11-16

**Authors:** Shay Menascu, Yulia Khavkin, Rina Zilkha‐Falb, Mark Dolev, David Magalashvili, Anat Achiron, Michael Gurevich

**Affiliations:** ^1^ Multiple Sclerosis Center, Sheba Medical Center Ramat‐Gan Israel; ^2^ Sackler School of Medicine Tel‐Aviv University Tel Aviv Israel

## Abstract

**Objective:**

To determine whether pediatric‐onset multiple sclerosis (POMS) and adults‐onset multiple sclerosis (AOMS) patients are different in initial disease severity and recovery and to investigate the associations with peripheral blood mononuclear cells (PBMCs) transcriptional profiles.

**Methods:**

Clinical and radiological severity of first and second relapses and 6‐month recovery were analyzed in 2153 multiple sclerosis (MS) patients and compared between POMS (onset at 8–18years old) and AOMS (onset at 19–40 years old) patients. PBMCs transcriptomes of 15 POMS and 15 gender‐matched AOMS patients were analyzed 6 months after the first relapse and compared to 55 age‐matched healthy controls. Differentially Expressed Genes (DEGs) with a false discovery rate ≤ 10% were evaluated using the Partek software.

**Results:**

POMS had increased Expanded Disability Status Scale (EDSS) score at first and second relapses, higher brain gadolinium‐enhancing T1‐lesions volume at first relapse, and more complete recovery after both relapses compared to AOMS. POMS patients, who recovered completely from the first relapse, were characterized by 19 DEGs that were mainly related to suppression of antigen presentation. Six upstream regulators of these genes were differentially expressed between pediatric and adult healthy controls. POMS patients, who showed no recovery from the first relapse, were characterized by 28 DEGs that were mainly associated with B‐cell activation. Five upstream regulators of these genes were differentially expressed between pediatric and adult healthy controls.

**Interpretation:**

POMS patients may have more severe first and second relapses than AOMS. However, most often, POMS have better recovery that may be attributed to PBMCs age‐related transcriptional profiles associated with antigen presentation and B‐cell activation.

## Introduction

Age is a significant factor in multiple sclerosis (MS), affecting the disease’s phenotype and prognosis. Pediatric‐onset MS (POMS), which occurs before 18 years of age, comprises 2‐5% of all MS cases.^1^, ^2^ POMS patients have more frequent polyfocal symptoms,^3^ higher relapse rate,^4^ higher rates of complete remission from initial relapse,^5^ and slower disease progression,^2^, ^6^, ^7^ in comparison to adult‐onset MS (AOMS) patients.

MS patients have a unique blood gene expression pattern related to activation of T‐cell expansion, inflammatory cytokines and integrins, and suppression of anti‐inflammatory cytokines and apoptosis.^8^, ^9^, ^10^ However, only a single study by Liguori et al. (2017)^11^ addressed the underlying molecular mechanism of POMS, comparing microRNA and mRNA gene expression of 19 POMS patients with that of 20 controls. The study revealed 13 deregulated microRNAs that were associated with autophagy and ATPase activity.

In this study, we aimed to determine whether disease‐modifying drugs (DMDs)‐free POMS and AOMS patients are different in initial disease severity and recovery and to investigate the associations with peripheral blood mononuclear cells (PBMCs) transcriptional profiles.

## Patients and Methods

### Study design

A retrospective cohort study of relapsing‐remitting multiple sclerosis (RRMS) patients followed at Sheba Medical Center, Multiple Sclerosis Center (MSC) between 2003 and 2018. The study was approved by Sheba Medical Center Institutional Review Board. Informed consent was obtained from each subject or legal guardian.

Clinical and radiological severity of first and second MS relapses and 6‐month recovery were analyzed and compared between POMS and AOMS patients. PBMCs transcriptomes in the subset of POMS and gender‐matched AOMS patients were analyzed 6 months after the first relapse and compared to age‐matched healthy controls.

### Patients

**Figure 1:**
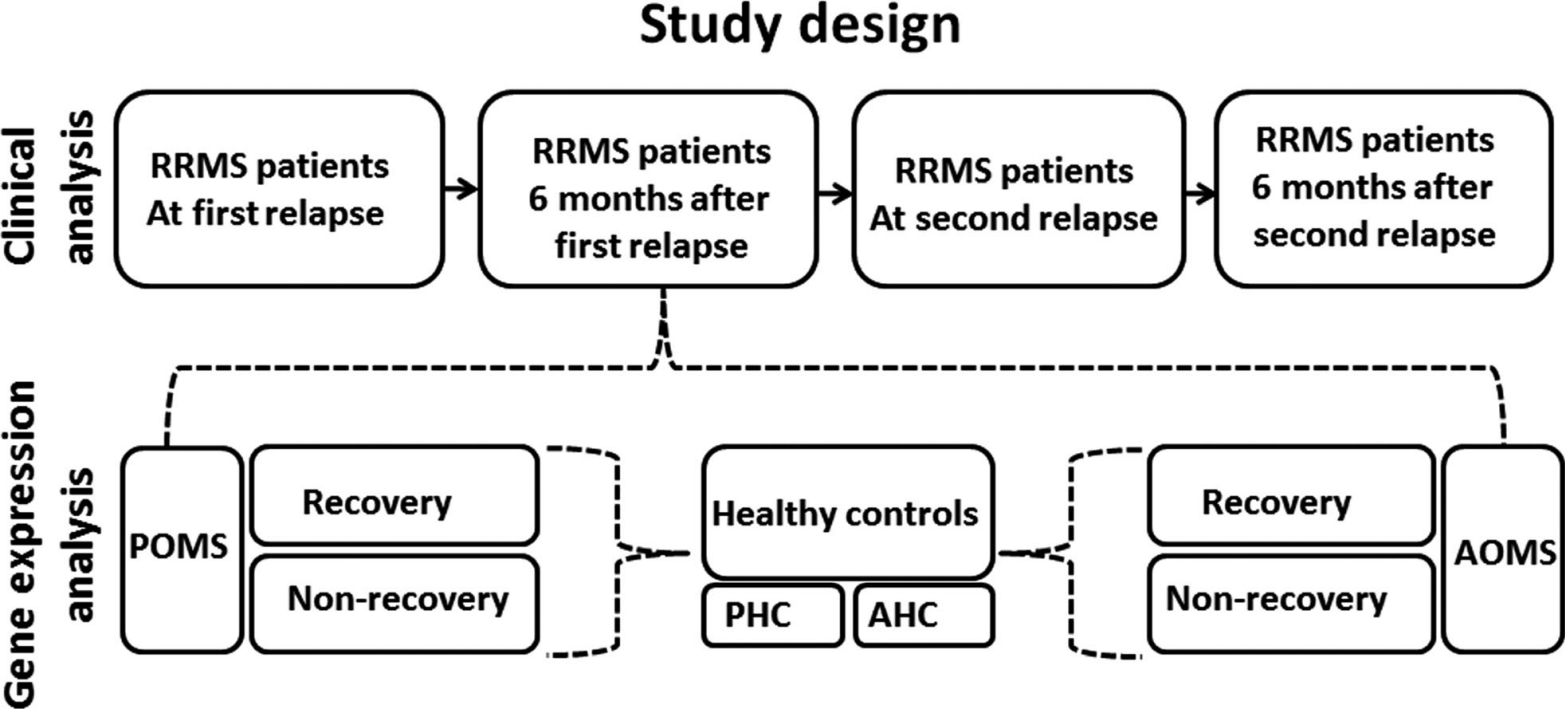
Schematic presentation of study design. Clinical parameters of DMDs‐free RRMS patients at first and second relapse and 6 months after each relapse were analyzed and compared between POMS and AOMS patients. Gene expression profile of PBMCs from first‐relapse‐recovered and nonrecovered POMS and AOMS patients were analyzed and compared to PBMCs obtained from healthy pediatric and adult controls. POMS – pediatric‐onset MS, AOMS – adult‐onset MS, PHC – pediatric healthy controls, AHC – adult healthy controls.

MS patients were selected according to the following inclusion criteria:

- POMS patients diagnosed according to the International Pediatric Multiple Sclerosis Study Group consensus definitions^12^ with disease onset at age ≤ 18 years;
- AOMS patients with disease onset at age 19‐40 years, diagnosed according to the 2010,^13^ 2017^14^ McDonald criteria;
- A neurological examination within 2–21 days from initial clinical symptoms of either first or second relapse;
- No DMDs treatment until 6 months after the second relapse;
- For transcriptional analysis, PBMCs obtained 6 months after the first relapse from a subset of POMS and AOMS patients that were matched in gender and recovery state. PBMC obtained from gender‐ and age‐matched healthy pediatric and adult subjects were used as healthy controls (HC), (Fig 1).

At first and second relapses, all patients were treated with high dose intravenous steroids. Patients that started DMDs treatment between the first and second relapses were excluded from the second relapse analysis.

### Clinical analysis

Clinical data were retrieved from the computerized MSC’s database. The results of neurological examinations and Expanded Disability Status Scale (EDSS) scores performed at either first or second relapse as well as 6 months after each relapse, were obtained. The relapses were defined as the onset of new or worsening of existing neurological symptoms, persisting for at least 48 hours to 21 days, with objective findings in a clinical neurological examination. The first relapse was referred as disease onset, therefore the pre‐relapse EDSS was defined as EDDS = 0. For the second relapse, pre‐relapse EDSS was assessed within 3 months before the relapse. Patients that did not have a pre–second relapse EDSS score were not included in the study.

Brain magnetic resonance imaging (MRI) data were acquired using a 3.0‐T MRI scanner (Signa; GE Healthcare, Chicago, IL, USA) with an axial T2‐weighted sequence (slice thickness 3.0 mm, no gap, field of view 240 mm, matrix 256x256, repetition time 5,100, echo time 80). Lesions were identified by an experienced radiologist and the number and volume of the T2 and hypointensive T1 gadolinium‐enchasing lesions were quantified using semiautomated segmentation analysis software (MSET‐1.9, Matlab‐12).

The following clinical parameters were used for comparing between POMS and AOMS patients: (a) EDSS at first relapse; (b) the number and volume of brain MRI T2 and hypointensive T1 gadolinium‐enchasing lesions; (c) the increase in EDSS at the first and second relapse calculated as the difference between peak EDSS during relapse and prerelapse EDSS; (d) the proportion of patients with EDDS increase ≥ 3.0 at the first and second relapse; (e) the number of impaired functional domains for both relapses; (f) residual disability 6 months after the first and second relapses, calculated as the difference between the 6‐month post‐relapse EDSS and the pre‐relapse EDSS; and (g) clinical recovery, defined as a reduction of ≥ 1.0 EDSS points 6 months postrelapse as compared to the peak of EDSS during relapse. Complete recovery was defined as a residual disability of 0 EDSS points, and incomplete recovery as a residual disability ≥ 1.0 EDSS points.

### Gene expression analysis

Transcriptional profiles of PBMCs obtained from POMS and AOMS patients at 6 months after the first relapse were analyzed using Affymetrix Inc. technology. This technology allows screening for a vast array of differentially expressed genes and is effective in discovering novel biological pathways. Briefly, PBMCs were separated on a ficolhypaque gradient. Total RNA was purified from PBMCs using TRlzol® (Invitrogen, Carlsbad, CA, USA) and Phase‐Look‐Gel columns (Eppendorf, Hamburg, Germany) including a DNase digestion step. RNA quality was assessed using Bio‐Rad Experion Automated Electrophoresis Station (Hercules, CA, USA) and quantified by fiber optic spectrophotometry using the Nanodrop ND‐1000. RNA yielding both an A260/A280 absorbance ratio greater than 2.0 and a 28s/18s rRNA ratio equal to or exceeding 1.8 was utilized. Double‐stranded cDNA was synthesized from 250 ng total RNA using the One‐Cycle cDNA Synthesis Kit, and in vitro transcription was performed with the GeneChip IVT Labeling Kit (both Affymetrix Inc., Santa Clara, CA, USA). cDNA was labeled with streptavidin phycoerythrin, and biotin‐labeled anti‐streptavidin phycoerythrin antibodies. The biotin‐labeled IVT‐RNA was hybridized to HG‐U133A‐2 arrays (Affymetrix, Santa Clara, CA, USA) containing ~ 22,000 gene transcripts corresponding to 14,500 well‐annotated human genes. The microarrays were then washed in a Gene‐Chip Fluidics Station 450 and scanned on a GeneArray‐TM scanner (G2500A; Hewlett Packard Palo Alto, California, USA) according to the standard Affymetrix Inc. protocol.

### Statistical analysis

#### Clinical analysis

Clinical and demographical data were presented as mean (95% confidence interval [CI]). Continuous variables were analyzed using two samples t‐test. The proportion of patients with first‐ and second relapse EDSS score ≥ 3.0 and the proportion of patients with incomplete recovery 6 months after relapses were examined by logistic regression. The odds ratio (OR) and 95% CI were calculated using a multivariate model. A p‐value of ≤ 0.05 was deemed statistically significant. Gender‐specific effects were also analyzed.

#### Gene expression analysis

Gene expression data were normalized using R, an open‐source software environment for statistical computing. Two methods of normalization were applied:

- Single Channel Array Normalization – a serial normalization method that increases signal‐to‐noise ratio within individual samples and decreases variation across samples.
- Combining Batch Normalization – a method for solving batch effects on data that allows combining results from different batches.

The Partek Genomics Software was used to evaluate Differentially Expressed Genes (DEGs) with False Discovery Rate (FDR) ≤ 10% after correction for multiple comparisons. DEGs between POMS and AOMS patients, for both, post‐relapse clinically recovered and non‐recovered patients were evaluated. Using the Ingenuity Pathway Analysis (IPA) software, potential upstream regulating genes for these DEGs were identified. These upstream regulators with their downstream target DEGs were tested for their contribution to the PBMCs transcriptional differences between healthy pediatric and adult controls.

The transcriptional data were deposited in NCBI's Gene Expression Omnibus (GEO), GEO Series accession number GSE146383 (https://www.ncbi.nlm.nih.gov/geo/query/acc.cgi?acc=GSE146383).

## Results

Of 3189 RRMS patients registered in the MSC’s database, 2153 patients (1500 females) fulfilled the inclusion criteria and were included in the analysis. The mean age at onset was 27.3 years (95% CI: 26.2–28.5 years). Of these 269 were POMS patients, with mean age at onset 15.6 years (95% CI: 15.4–15.9 years), Female/Male (F/M) ratio 2:1, and 1884 were AOMS patients with the mean age at onset 29.0 years (95% CI: 28.7–29.3 years), F/M ratio 2.3:1.

At first relapse, neurologic disability by mean EDSS was 2.5 (95% CI: 2.5–4.7), the number of gadolinium‐enhancing T1 brain MRI lesions and T2 brain MRI lesions was 1.2 (95% CI: 1.0–1.3) and 15.9 (95% CI 13.9–17.9), respectively, volume of gadolinium‐enhancing T1 brain MRI lesions and volume of brain MRI T2 lesions was 0.2 cm^3^ (95% CI: 0.2–0.3 cm^3^) and 2.9 cm^3^ (95% CI: 2.3–3.2 cm^3^), respectively. Approximately 98 % of patients had polysymptomatic functional system involvement at clinical presentation with the mean number of impaired functional domains 1.4 (95% CI: 1.0–2.5).

Of the 2153 RRMS patients analyzed at first relapse, 998 patients received DMDs treatment (glatiramer‐acetate [17%], immunoglobulins [15%], interferons [58%], natalizumab [3%] dimethyl‐fumarate [7%]) before the second relapse and were excluded from second relapse analysis. The rest DMDs‐naive patients (*n* = 1155) were subjected to second relapse analysis. DMDs treatment was initiated in these patients 3.2 years (95% CI: 2.8–3.6 years) after the second relapse.

### The Difference between POMS and AOMS patients in first relapse severity and recovery

**Figure 2:**
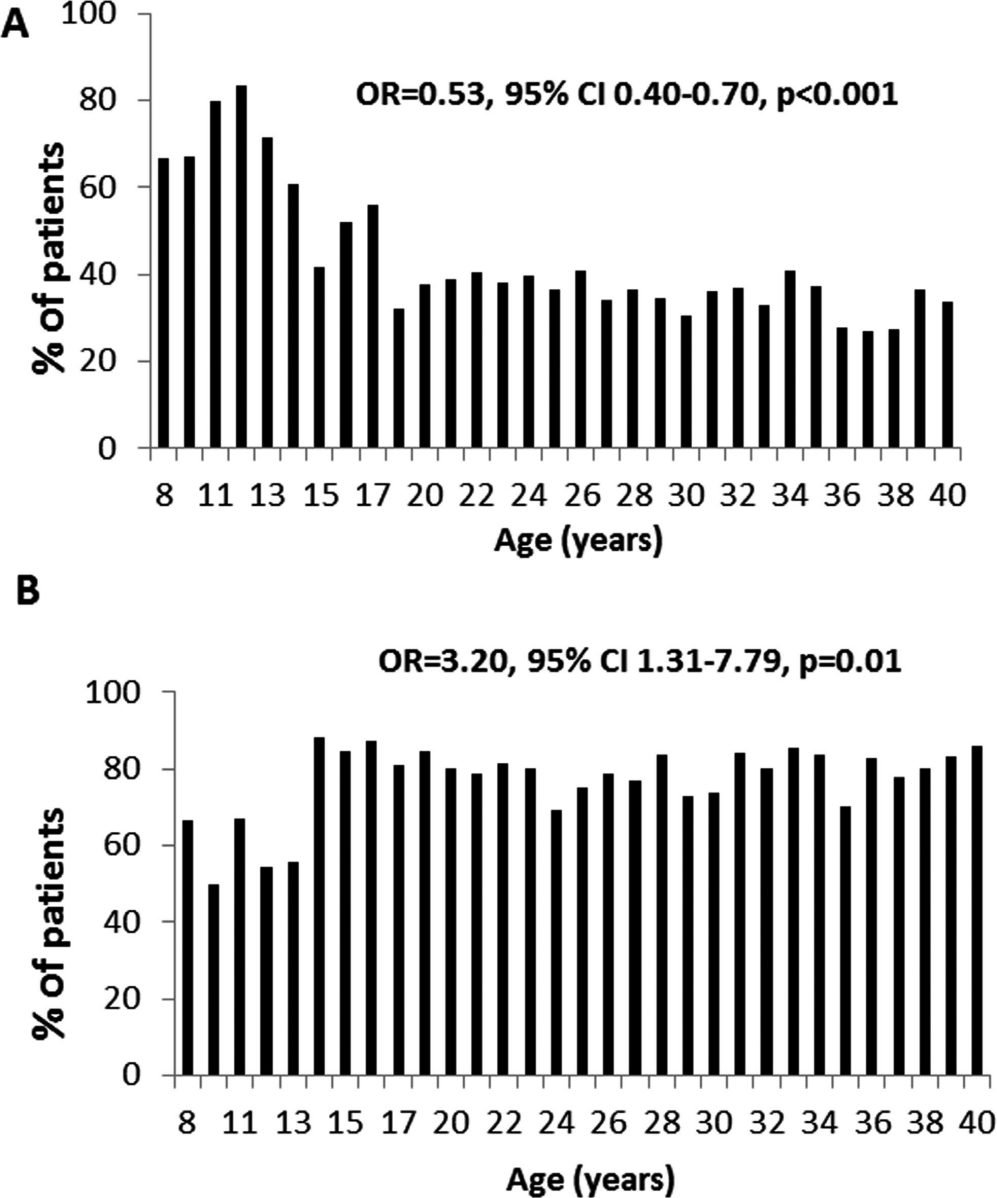
Severity and recovery of first MS relapse in POMS and AOMS patients. (A) The proportion of MS patients with EDSS scores ≥ 3.0 at first MS relapse by age at first relapse. Pediatric MS onset is associated with a higher proportion of patients with more severe disability (*P* < 0.001). (B) The proportion of MS patients with residual disability (EDSS ≥ 1.0) 6 months after the first MS relapse by age at onset. Pediatric MS onset is associated with lower residual disability (*P* = 0.01).

POMS patients had a more severe first relapse compared with AOMS patients as indicated by a significantly higher mean EDSS score (2.7 [95% CI: 2.6–4.8] vs. 2.0 [95% CI: 1.9‐4.2], (*P* = 0.004), higher proportion of patients with EDSS ≥ 3.0 (OR = 0.53, [95% CI: 0.40–0.70]), *P* < 0.001, (Fig. 2A) and greater number of impaired functional domains (OR = 0.02, [95% CI: 0.01–0.4]), *P* = 0.01.

Comparison of first‐relapse EDSS between males (*n* = 647) and females (*n* = 1506) showed that POMS was associated with higher proportion of EDSS ≥ 3.0 in both subgroups (OR = 0.61, 95% CI: 0.46–0.81 and OR = 0.51, 95% CI: 0.34–0.75, respectively), *P* < 0.001.

Mean EDSS was higher in male POMS compared to male AOMS patients (2.7 [95% CI: 2.6–2.9] vs. 2.3 [95% CI: 2.2–2.3], respectively), *P* = 0.02 but not in females (2.6 [95% CI: 2.4–2.8] and 2.5 [95% CI: 2.5–2.6], respectively), *P* = 0.4.

Six months after the first relapse, the mean EDSS score was 1.3 (95% CI: 1.2–1.4) and 79.2% of patients had residual disability ≥ 1.0 EDSS point. POMS patients were associated with a lower proportion of patients with residual disability ≥ 1.0 points compared to AOMS (OR = 3.20, 95% CI: 1.31–7.79), *P* = 0.01, Figure 2B.

The analysis of the effect of first‐relapse EDSS severity on recovery 6 months later showed that, as expected, EDSS ≥ 3.0 at relapse was strongly associated with a higher proportion of patients with incomplete recovery 6 months later (OR = 12.8, 95% CI: 8.5–19.4), *P* < 0.001. Interestingly, when the same analysis was performed separately on POMS and AOMS patients, higher EDSS at relapse was associated with poor recovery only AOMS patients (OR = 4.7, 95% CI: 3.2–6.8, *P* < 0.001), whereas in POMS patients good recovery was observed independently from EDSS at relapse (OR = 1.1, 95% CI: 0.7–2.9, *P* = 0.16).

### The Difference between POMS and AOMS patients in second relapse severity and recovery

On average, the second relapse occurred significantly earlier in POMS patients compared to AOMS patients: 0.9 years (95% CI: 0.8–1.1 years) vs. 3.5 years (95% CI: 3.2–3.8 years) after first relapse, respectively, *P* = 0.01. Mean EDSS before the second relapse was similar in both populations: 1.4 (95% CI: 1.3–1.5) for POMS and 1.3 (95% CI: 1.3–1.5) for AOMS patients. Although the mean EDSS‐increase at second relapse was greater in POMS compared to AOMS patients: 1.5 (95% CI: 1.2–1.8) vs. 1.2 (95% CI: 1.2–1.6), *P* = 0.05, the proportion of patients with EDSS increase ≥ 3.0 was similar in both groups (45% and 41%, respectively, *P* = 0.5). The polysymptomatic functional system presentation was 46% in the POMS patients and 49% in the AOMS populations with a mean of 1.7 (95% CI: 1.5–1.9) impaired functional domains in both groups.

AOMS was also associated with a higher proportion of incomplete recoveries after the second relapse (OR = 1.4, 95% CI: 1.04–1.9), *P* = 0.02. This effect was significant only in males but not in females (OR = 1.9, 95% CI 1.1–3.2, *P* = 0.01 and OR = 1.2, 95% CI 0.8–1.7, p = 0.3, respectively). The proportion of AOMS patients with incomplete recovery demonstrated a tendency for an association with EDSS increase at second relapse (OR = 0.67, 95% CI 0.44–1.03, *P* = 0.06).

In order to test the hypothesis that clinical presentation of second relapse is more affected by age at second relapse than by age of onset, second relapse severity and recovery were compared between MS patients with pediatric age (<18 years old) and adult age (19–40 years old) at time of second relapse. The results demonstrated that the proportion of patients with an EDSS increase ≥ 3.0 at second relapse and the proportion of patients with incomplete recovery were not significantly different between pediatric and adult MS patients (OR = 0.74, 95% CI: 0.50‐1.11, *P* = 0.2 and OR = 1.30, 95% CI: 0.72–2.34, *P* = 0.3, respectively). Next, we excluded 109 POMS patients who had a second relapse at age >18 years, and AOMS with a second relapse at age > 40 years, leaving 1046 patients whose age status did not change during the follow‐up period. In this subanalysis, in the POMS patients, the proportion of patients with EDSS ≥ 3.0 at second relapse was higher (OR = 0.26, 95% CI: 0.17–0.40), *P* < 0.01) and the proportion of patients with incomplete recovery was lower (OR = 1.77, 95% CI 0.98–3.2, *P* = 0.05). These results suggest that second relapse severity and recovery are more associated with the age of MS onset.

In addition, by applying ANOVA model to compare second relapse severity between POMS patients who remained pediatric at second relapse, POMS who had second relapse at adult age, and AOMS, we have confirmed that EDSS at second relapse more significantly associated with age at onset (*P* = 0.02) than with age of second relapse (*P* = 0.2).

### Effect of gender on first‐ and second relapse severity and recovery

No association was noted between gender and severity of first and second relapse (OR = 1.04, 95% CI: 0.85‐1.27, *P* = 0.7, OR = 1.0, 95% CI: 0.79–1.33, *P* = 0.8, respectively). Gender was also not associated with the proportion of patients showing incomplete recovery 6 months after the first two relapses (OR = 0.98, 95% CI: 0.74–1.3, *P* = 0.9 and OR = 0.85, 95% CI: 0.58–1.25, *P* = 0.4, respectively).

### Age‐related PBMCs transcriptional profiles associated with recovery from first MS relapse

Fifteen POMS patients, mean age 15.5 years (95% CI: 14.8–16.2 years), F/M ratio 1.1:1, mean EDSS at first relapse 3.1 (95% CI: 2.6–3.5), and 15 AOMS patients, mean age 31.9 years (95% CI: 28.9–34.8 years), F/M ratio 1.1:1, mean EDSS at first relapse 2.6 (95% CI: 2.1‐3.1), donated blood samples for gene expression analysis. An age‐ and gender‐matched healthy cohort (*n* = 55) consisting of 46 adults, mean age 34.4 years (95% CI: 32.6–36.3 years), F/M ratio 1.1:1 and nine pediatric subjects, mean age 13.9 years (95% CI: 11.6–16.3), F/M ratio 1.2:1 were enrolled.

PBMCs transcriptional profiles were compared between POMS (*n* = 8) and AOMS (*n* = 6) patients who showed a clinical recovery ≥ 1.0 EDSS with mean residual disability of 1.0 (95% CI: 0.6–1.6) and 1.5 (95% CI: 0.8–2.3), respectively, 6 months after relapse. POMS (*n* = 7) and AOMS (*n* = 9) patients whose EDSS did not change 6 months after relapse and did not show clinical recovery were also compared.

Comparison between PBMCs gene expression profiles of POMS and AOMS patients that showed clinical recovery after their first relapse revealed 19 DEGs, that were mainly associated with underexpression of antigen‐presenting mechanisms (*P* = 1.36E‐3). These DEGs included genes like HLA‐DQA1, HLA‐DQB1, END1, and RAB27A, as well as more highly expressed apoptosis‐related genes such as ARHGEF12 (Table 1).

**Table 1:** DEGs between post‐relapse recovered POMS and AOMS patients.

DEGs Name	DEGs Title	Fold/Change Direction
HLA‐DQB1	major histocompatibility complex, class II, DQ beta 1	Down
HLA‐DQA1	major histocompatibility complex, class II, DQ alpha 1	Down
MID2	midline 2	Down
NAP1L3	nucleosome assembly protein 1‐like 3	Down
EDN1	endothelin 1	Down
OGG1	8‐oxoguanine DNA glycosylase	Down
CACNA1I	calcium channel, voltage‐dependent, T type, alpha 1I subunit	Down
HSPA4L	heat shock 70kDa protein 4‐like	Up
WSB2	WD repeat and SOCS box containing 2	Up
BYSL	bystin‐like	Up
RAB27A	RAB27A, member RAS oncogene family	Up
GPC4	glypican 4	Up
ARHGEF12	Rho guanine nucleotide exchange factor (GEF) 12	Up
KCTD14	potassium channel tetramerization domain containing 14	Up
MSI1	musashi RNA‐binding protein 1	Up
ENPP4	ectonucleotide pyrophosphatase/phosphodiesterase 4	Up
CELP	carboxyl ester lipase pseudogene	Up
DBI	diazepam‐binding inhibitor	Up
MCF2L2	MCF.2‐cell line–derived transforming sequence‐like 2	Up

DEGs, Differentially Expressed Genes.

**Figure 3:**
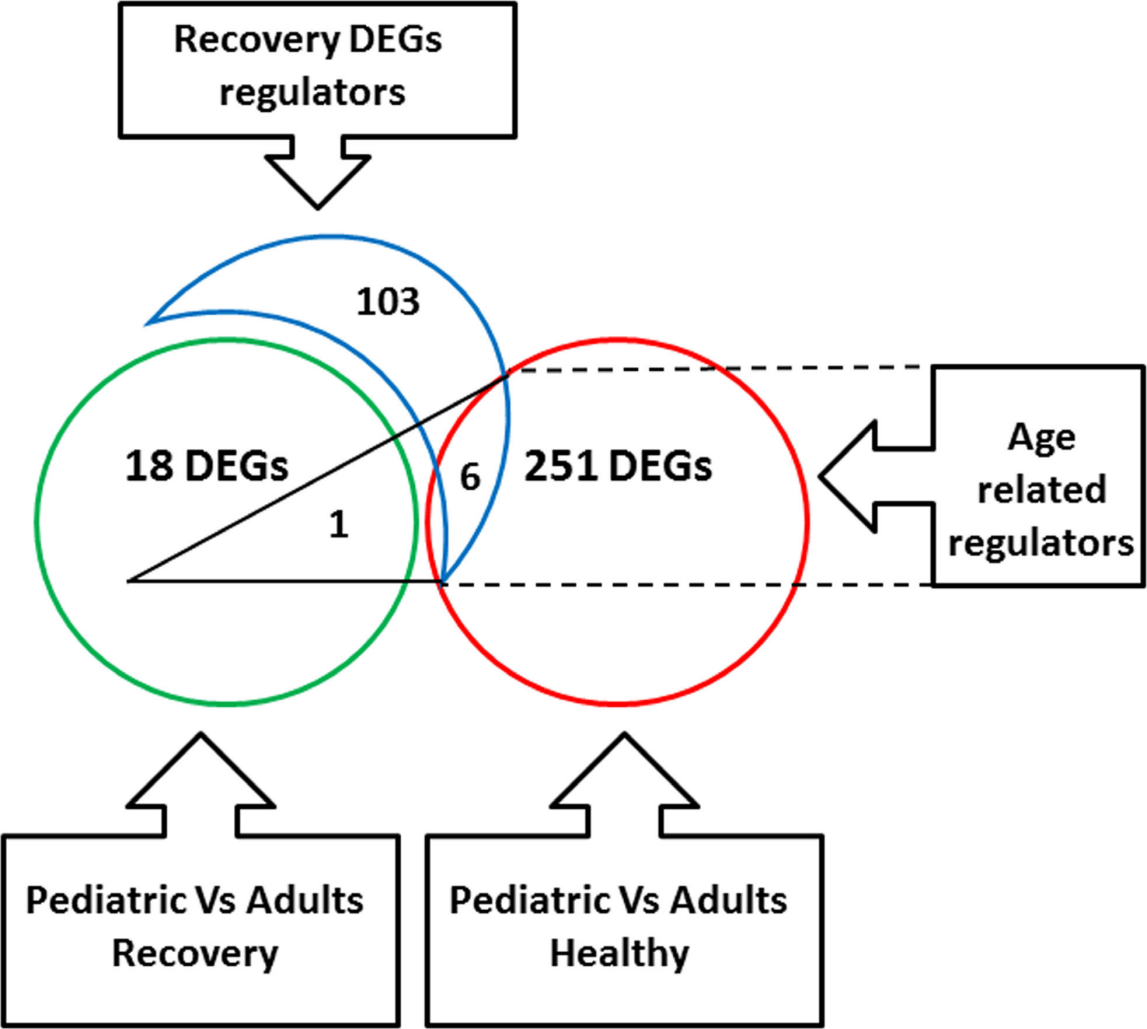
Venn diagram of numeric gene expression results of post‐relapse recovered MS patients. Nineteen DEGs (green circle) differentiate between post‐relapse recovered POMS and AOMS patients. These DEGs are regulated by 109 upstream regulating genes, (blue crescent). 257 DEGs differentiate between healthy pediatric and adult controls (red circle). Of these six were included in the 109 upstream regulating genes, and involved in regulating one of 19 DEGs that differentiated between post‐relapse recovered POMS and AOMS patients.

**Figure 4:**
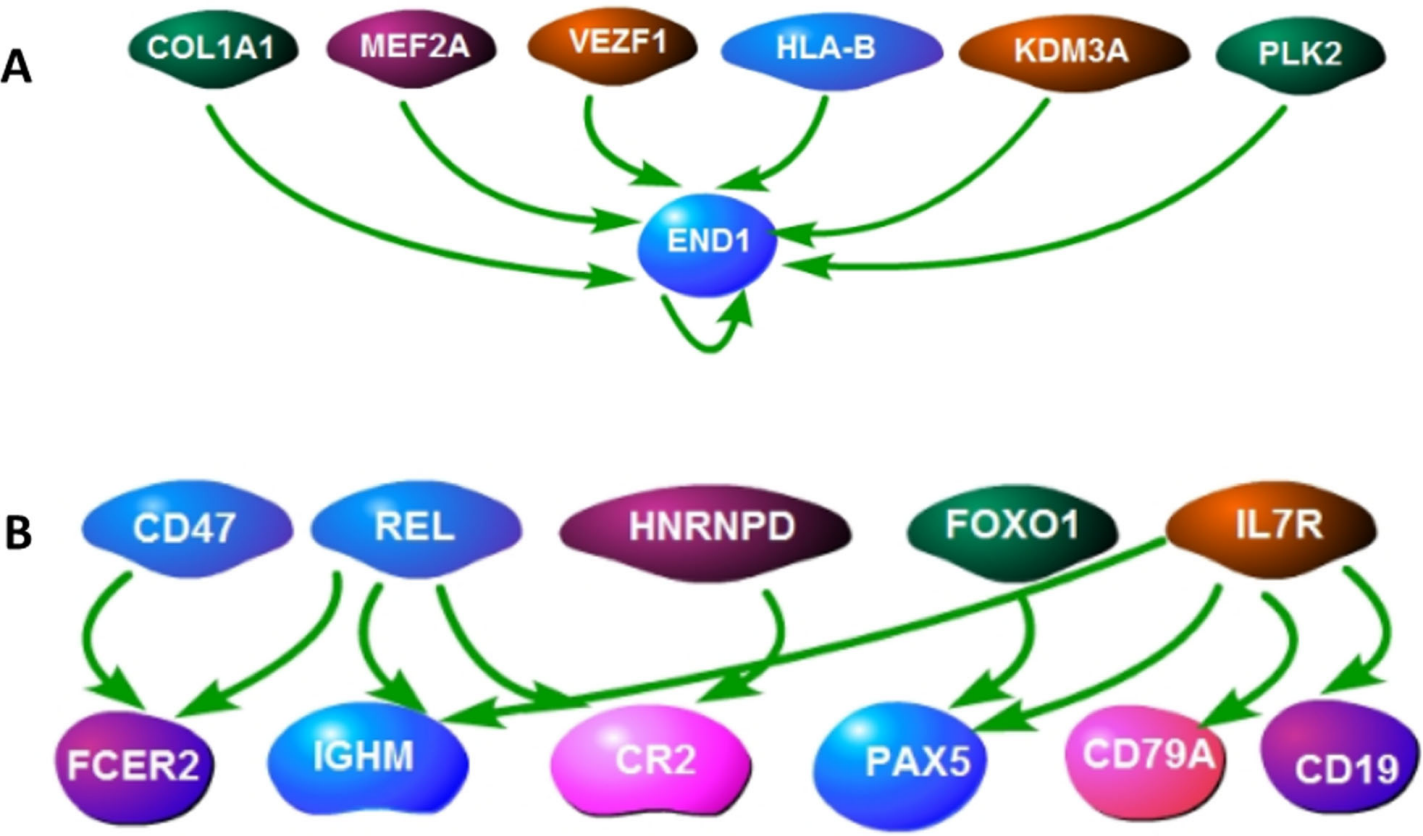
Age‐related upstream regulators of DEGs between POMS and AOMS patients. (A) Age‐related upstream regulators of DEGs in post‐relapse recovered POMS and AOMS patients. (B) Age‐related upstream regulators of DEGs in post‐relapse non‐recovered POMS and AOMS patients. COL1A1 – collagen‐type I alpha 1 chain, MEF2A – myocyte enhancer factor 2A, VEZF1 – Vascular Endothelial Zinc Finger 1, KDM3A – Lysine Demethylase 3A, PLK2 – Polo Like Kinase 2, END1 – Endothelin 1, CD47 – Leukocyte Surface Antigen CD47, REL – REL proto‐oncogene, HNRNPD – Heterogeneous Nuclear Ribonucleoprotein D, FOXO1 – Forkhead Box O1, IL7R – Interleukin 7 Receptor, FCER2 – Fc Fragment Of IgE Receptor II, IGHM – Immunoglobulin Heavy Constant Mu, CR2 – Complement C3d Receptor 2, PAX5 – Paired Box 5, CD79A – B‐Cell Antigen Receptor Complex‐Associated Protein Alpha Chain, CD19 – B‐Lymphocyte Antigen CD19.

The analysis of upstream regulators of these 19 DEGs revealed 109 potential regulators (Table S1). To test the hypothesis that these regulators and their 19 downstream DEGs could be part of the transcriptional difference between healthy pediatric and adult subjects, PBMC expression profiles of these two healthy groups were compared, revealing 257 DEGs (Table S2). Of the 109 potential upstream regulators mentioned above, six were differentially expressed (*P* < 0.004) between healthy pediatric and adult controls: COL1A1, MEF2A, VEZF1, HLAB, KBM3A, PLK2 (Fig. 3). All six upstream regulators could affect the one downstream END1gene (Fig. 4A).

**Figure 5:**
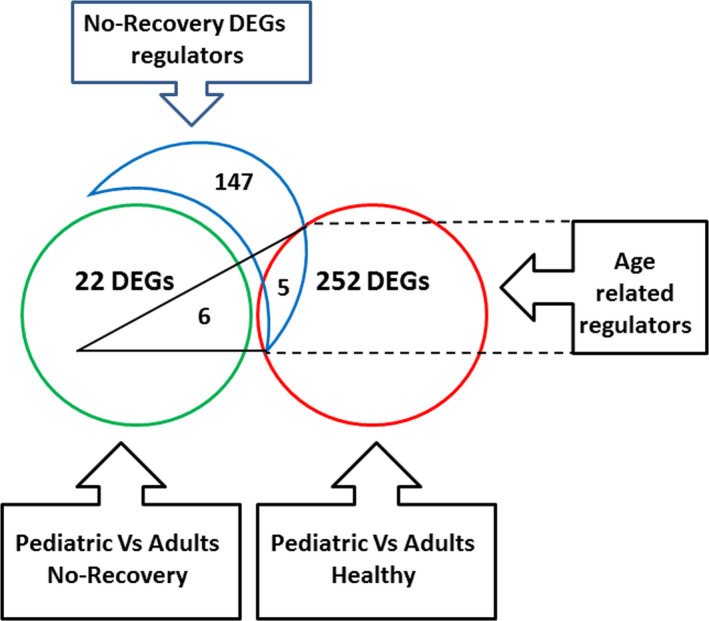
Venn diagram of numeric gene expression results of post‐relapse non‐recovered MS patients. A total 28 DEGs (green circle) differentiated between post‐relapse non‐recovered POMS and AOMS patients. These DEGs are regulated by 152 upstream regulators (blue crescent). 257 DEGs differentiate between healthy pediatric and adult subjects (red circle).Of these, five were included in the 152 upstream regulating genes and involved in regulating six of 28 DEGs that differentiated between post‐relapse non‐recovered POMS and AOMS patients.

Comparison between PBMC expression profiles of POMS and AOMS patients that showed no recovery after their first relapse revealed 28 DEGs. These DEGs are mainly associated with activation of B‐cell development (*P* = 3.39E‐7), B‐cell proliferation (*P* = 1.70E‐12), and B‐cell receptor signaling (*P* = 7.0E‐08) and included genes such as CD19, CD22, MS4A1, BCL7A, IGHM, IGHD, CD79A, BLNK, FCER2, CR2, PAX5, IL4R, and STAP1 (Table 2). In addition, 152 potential upstream regulators were identified for these 28 DEGs (Table S3) of which five (CD47, REL, HNRNPD, FOXO1, and IL7R) were differentially expressed (*P* < 0.001) between healthy pediatric and adult controls (Fig. 5). These upstream regulators could affect CD19, CD79, IGHM, PAX5, FCER2, and CR2 DEGs which were differentially expressed between POMS and AOMS patients that showed no recovery after first relapse (Fig. 4B).

**Table 2:** DEGs between post‐relapse non‐recoverd POMS and AOMS patients.

DEGs Name	DEGs Title	Fold/Change Direction
C16orf3	chromosome 16 open reading frame 3	Down
APH1B	anterior pharynx defective 1 homolog B (C. elegans)	Down
SQRDL	sulfide quinone reductase‐like (yeast)	Down
SAMSN1	SAM domain, SH3 domain, and nuclear localization signals 1	Down
HTATIP2	HIV‐1 Tat interactive protein 2, 30kDa	Down
ENY2	enhancer of yellow 2 homolog (Drosophila)	Down
ACSL1	acyl‐CoA synthetase long‐chain family member 1	Down
COX7B	cytochrome c oxidase subunit VIIb	Down
FPR2	formyl peptide receptor 2	Down
CD19	CD19 molecule	Up
BCL7A	B‐cell CLL/lymphoma 7A	Up
IGHM	immunoglobulin heavy constant mu	Up
CD22	CD22 molecule	Up
IGHD	immunoglobulin heavy constant delta	Up
MS4A1	membrane‐spanning 4‐domains, subfamily A, member 1	Up
PAWR	PRKC, apoptosis, WT1, regulator	Up
ABCB4	ATP‐binding cassette, subfamily B (MDR/TAP), member 4	Up
FADS3	fatty acid desaturase 3	Up
PCDH9	protocadherin 9	Up
CD79A	CD79a molecule, immunoglobulin‐associated alpha	Up
MARCH3	membrane‐associated ring finger (C3HC4) 3	Up
TCF4	transcription factor 4	Up
BLNK	B‐cell linker	Up
IL4R	interleukin 4 receptor	Up
FCER2	Fc fragment of IgE, low affinity II, receptor for (CD23)	Up
CR2	complement component (3d/Epstein Barr virus) receptor 2	Up
TCL1A	T‐cell leukemia/lymphoma 1A	Up
STAP1	signal transducing adaptor family member 1	Up

DEGs, Differentially Expressed Genes.

## Discussion

We evaluated the role of pediatric and adult ages of MS onset in the heterogeneity of the first and second MS relapse severity and recovery in a large cohort of DMDs‐free RRMS patients with disease onset of 8–40 years. Our findings show, that although POMS patients may have more severe first and second relapse than AOMS, their recovery from relapse is often better and independent of relapse severity, as demonstrated by lower residual disability six months later. An association between relapse severity and less efficient recovery was only observed in the AOMS patients. Age‐related underexpression of PBMCs genes involved in antigen‐presenting functions or to overexpression of B‐cell associated genes is attributed to the difference noted between POMS and AOMS with regard to the recovery from acute MS relapse.

The difference between POMS and AOMS features has been reported in previous publications.^15^, ^16^ Our clinical observation is in complete agreement with several retrospective American and European reports that concluded that POMS patients have more severe onset but better recovery and slower disability accumulation as compared with AOMS patients.^5^, ^17^, ^18^, ^19^

Previous studies have analyzed relapse recovery in patient cohorts with variable treatments and clinical presentations. Moreover, the intervals between relapse and postrelapse EDSS assessment ranged from a single month to year, leading to inconsistent results in relation to the duration of postrelapse recovery and the level of postrelapse residual disability.^20^, ^21^, ^22^ Studies with relatively short intervals between relapse and postrelapse EDSS analysis may overestimate the residual disability level, thus the patient not have reached the end of their recovery. In contrast, studies with relatively large intervals for post‐relapse EDSS assessments may have had an EDSS bias associated with disease progression. We chose to measure recovery 6 months after relapse following studies by Hirst et al. (2008),^20^ Koch‐Henriksen et al. (2019),^21^ and Novotna et al. (2015),^23^ that have suggested that most patients recover 3–6 months after the relapse. For example, Hirst et al. (2008)^20^ measured post‐relapse EDSS for 36–496 days, and reported that recovery changes took place until 180 days post‐relapse.

In order to clarify whether the age of onset or age at second relapse have a more robust effect on second relapse outcome, we compared the second relapse clinical parameters only in POMS patients that were younger than 18 years during follow‐up, to chose of AOMS patients who were younger than 40 years during follow up. The obtained results support the hypothesis that second relapse severity and recovery depend mostly on the age of MS onset.

According to our results, 79.2% of patients had an incomplete recovery after the first relapse. This percentage is greater than the previously reported range of 34% by Leone at al. (2008),^24^ 42% by Lublin et al. (2003),^22^ 49.9% by Hirst et al. (2008),^20^ 53.3% by Kantarci et al. (2020),^25^ 54.5% by Vercellino et al.(2009),^26^ and 59.6% by West et al. (2006).^27^ Directly comparable studies are limited. West et al. (2006)^27^ analyzed the recovery of 186 MS patients following their first demyelinating event. These patients had various EDSS levels: 41.9% had mild (EDSS 0‐1.5), 44.1% had moderate (EDSS 2.0‐2.5), and 14% had severe (EDSS> 2.5). Incomplete recovery was reported for 59.6% of patients that is somewhat lower than our observation of 79.2%. Our finding may be explained by the fact that we included patients with more severe disease onset (EDSS 2.0 −3.0), whereas in West et al. study (2006)^27^ more than 40% of patients had onset EDSS between 0 and 1.5. This may have led to an underestimation of the first relapse residual disability in West et al. study. Notably, the authors reported that among those with severe onset, only 23.1% had complete recovery, meaning that 76.9% of patients had different levels of residual disability which is in complete agreement with our findings. A similar trend was reported by Lublin et al. (2003)^22^ whereby 37.5 % of included patients had no worsening of EDSS score during relapse and when residual disability was calculated in subgroups that experienced EDSS worsening during relapse, the rate of post‐relapse residual disability increased from 42% to 57%. Therefore, even if relapse severity is based on different definitions, our study and others show that higher severity of the initial relapse is associated with higher post‐relapse residual disability.^22^, ^24^, ^27^, ^28^ In addition, as only DMDs‐free patients were included in our study, this could contribute to the relatively higher incomplete relapse recovery.

The involvement of antigen presentation, in recovery mechanism is not surprising, since these processes are known to play a role in MS disease course and in MS susceptibility presented by polymorphism of HLA‐DQB1,^29^, ^30^, ^31^ IL4R,^32^ and HSP970^33^, ^34^ genes. A review by Anagnostouli et al. (2018)^35^ concluded that while HLA‐DRB1*1501 is clearly a risk factor for both POMS and AOMS patients, the results regarding the association between HLA‐DRB1 variability and age of MS onset are conflicting: some studies describe HLA‐DRB1*1501 as being associated with an earlier onset age, whereas others claim that there is no correlation between HLA‐DRB1*1501 and age. Of note, the HLA‐DRB1*1501 allele, which is known to increase the risk for developing MS^36^, ^37^, is associated with a high expression level of HLA‐DQB1.^38^ It is therefore possible that the underexpression of this gene in POMS patients compared to AOMS could be also a result of a lower prevalence of this allele in this subgroup.

In order to distinguish the role of pediatric‐onset and adult‐onset age in the MS post‐relapse recovery process, we performed an Ingenuity^®^ Knowledge Base Upstream Regulator analysis. We identified upstream regulators of DEGs that differentiate between post‐relapse recovered and non‐recovered POMS and AOMS patients. Some of these upstream regulators were also differentially expressed between healthy adult and pediatric controls. These findings suggest that age could contribute to the difference in recovery potential between POMS and AOMS patients.

Interestingly, all upstream regulators of DEGs between post‐relapse recovered POMS and AOMS affect END1 gene expression, which was found to be underexpressed. END1 has proinflammatory function through activation of NF‐κB and expression of cytokines such as TNF‐α, IL‐1, and IL‐6.^39^ Specifically, it plays a role in antigen presentation: dendritic cells, the major antigen‐presenting cells of the adaptive immune system, express END1.^40^ END1 is also overexpressed in other autoimmune diseases such as vasculitis.^41^

The age‐related upstream regulators of DEGs between post‐relapse non‐recovered POMS and AOMS patients affect genes that are associated with B‐cell activation (CD19, CD79A, IGHM, and PAX5). Correlation of incomplete recovery with age‐dependent B‐cell gene expression is one of the interesting observations of our study. In MS B cells are employed as precursors of antibody‐secreting cells, driving inflammation through the production of cytokines and chemokines or as antigen‐presenting cells by expressing MHC class II molecules, upregulating costimulatory molecules, and presenting their cognate antigen for activation of antigen‐specific T cell.^42^, ^43^ Despite the known efficacy of B‐cell depleting therapies, data on differences in B‐cell‐associated autoimmune mechanisms in pediatric and adults MS onset patients are limited. One study reported that circulating anti‐myelin oligodendrocyte glycoprotein (anti‐MOG) antibodies was strongly correlated with the age of MS onset.^44^ These antibodies, which can fix the complement and bind to Fc causing damage to oligodendrocytes were present in 38.7% of pediatric patients whose MS disease onset occurred at under 10 years of age, in 14.7% of patients whose disease onset occurred at 10–18 years of age, and only in 4% of AOMS patients. One can speculated that overexpression of B‐cell‐related genes, as we have observed in a subset of post‐relapse non‐recovered POMS patients, could be associated with such anti‐MOG reactivity.

Better recovery, regardless of relapse severity, in POMS patients in contrast to the correlation between recovery and relapse severity in AOMS patients could be attributed to neuroplasticity, or to the ability of the central nervous system to recover from damage through the structural and functional changes,^3^, ^45^ including myelin formation, which is most remarkable in children but continues up to the age of 18 years.^46^, ^47^ The significant effect of age on recovery has been well studied in animal models, and seems to be related to deterioration of signaling of oligodendrocyte precursors (OPC) with aging.^48^, ^49^, ^50^ It was demonstrated that aged OPC have a diminished ability to differentiate due to age‐related DNA damage and mitochondrial dysfunction and indirectly affected by the reduced ability of aged supporting cells (macrophages, microglia, and astrocytes) to produce proremyelination factors and remove myelin debris.

Some limitations must be considered in our study. First, our analyses were confined to the natural history of the first and second MS relapses in DMDs‐free MS patients; therefore, our findings cannot be applied to subsequent or DMDs‐treated relapses. Second, during relapses, all patients were treated with high‐dose intravenous steroids for five subsequent days. The possibility that steroids treatment interferes with natural recovery mechanisms should be considered. Third, we used EDSS as a measure of MS relapse severity and recovery. Some argue that the lower values of the EDSS measure impairments based on the neurological examination, whereas the higher values of the EDSS focus on walking ability.^51^, ^52^ Therefore, changes between steps on the scale are unequal and some functions such as cognition, energy level, pain or spasticity are not assessed, so that clinical outcome assessed by EDSS may have a lower limit of responsiveness. Lastly, although the clinical differences between POMS and AOMS patients were demonstrated in a large cohort of RRMS patients, only a relatively small number of patients donated blood for gene expression analysis. Nevertheless, the extensive analysis of clinical and transcriptional changes in all eligible patients enabled us to reveal the differences between POMS and AOMS patients in relapse‐related clinical outcomes and to suggest underlying biological mechanisms that could explain the clinical observations.

In conclusion, POMS patients may suffer from more severe neurological disability at first and second relapses; they more often show better recovery and lower residual disability. Short‐term clinical outcomes of MS may be modulated by age‐related differences in PBMCs transcriptional profiles. Improved recovery among POMS patients may be associated with the underexpression of antigen‐presenting mechanism, whereas the lack of recovery may be associated with overexpression of B‐cell‐related genes.

## Conflict of Interest

There are no financial disclosures relevant to the manuscript (no other study funding) and no conflicts of interest.

## Funding Information

No funding information is provided.

## Supporting information


**Table S1.** Upstream regulators of 19 recovery associated DEGs. Upstream regulators analysis revealed 109 potential regulators for 19 DEGs between POMS and AOMS patients that showed clinical recovery after their first relapse. DEGs – Differentially Expressed Genes, POMS – Pediatric Onset Multiple Sclerosis, AOMS – Adult Onset Multiple Sclerosis

**Table S2.** DEGs between pediatric and adult healthy controls 257 DEGs between healthy pediatric (age ≤ 18 years old) and adults (age 19–40 years old) controls. DEGs – Differentially Expressed Genes

**Table S3.** Upstream regulators of 28 DEGs associated with no recovery. Upstream regulators analysis revealed 152 potential regulators for 28 DEGs between POMS and AOMS patients that showed no clinical recovery after their first relapse. DEGs – Differentially Expressed Genes, POMS – Pediatric Onset Multiple Sclerosis, AOMS – Adult Onset Multiple Sclerosis
