# Supplementary material for: Clinical and transcriptional recovery profiles in pediatric and adult multiple sclerosis patients
Source: Ann Clin Transl Neurol. 2020 Nov 16;8(1):81–94. doi: 10.1002/acn3.51244 (PMC7818128; doi:10.1002/acn3.51244)
Supplement: Supplementary file 1 — Table S1. Upstream regulators of 19 recovery associated DEGs. Upstream regulators analysis revealed 109 potential regulators for 19 DEGs between POMS and AOMS patients that showed clinical recovery after their first relapse. DEGs – Differentially Expressed Genes, POMS – Pediatric Onset Multiple Sclerosis, AOMS – Adult Onset Multiple Sclerosis [file ACN3-8-81-s001.docx]

**Supplementary Table 1. Upstream regulators of 19 recovery associated DEGs.**

| **Upstream regulators** | **Target molecules** |
| --- | --- |
| Arnt-Hif1a  NADPH oxidase  hemoglobin  HLA-B27  IL27  CSF3  IFNG  NCF1  CASZ1  FUT9  POR  LIPE  DIRAS3  NOS1  ICMT  PARP9  CRHR1  BDKRB1  AGTR1  BDKRB2  Pkg  Angiotensin II receptor type 1  PRKAA  Gata  Relaxin  Atrial Natriuretic Peptide  Trk Receptor  AURK  Endothelin  MIR320  TLR7/8  BDNF  PGF  VEGFA  TRPV1  CACNA2D1  Cacnb1  NEK7  STK40  MKNK1  ROCK1  PLK2  PLK4  NR0B1  PPARA  NR3C2  AR  miR-128-3p  mir-135  mir-199  COL1A1  TRG  ARHGDIG  RASSF5  HLA-DQB1  MADD  SMC3  ELMO1  INSIG1  TBC1D10A  RGD1560225  PAEP  BIRC5  ANLN  DYSF  DCN  BAG1  HRG  NPPA  RCE1  ECE1  DOT1L  SGPP2  KDM3A  STAT3  STAT1  MXD1  CIITA  HAND1  HOXA10  NFYB  SIAH2  RFXAP  HIF3A  HDAC7  NFYA  CTCF  BCL3  ZBTB32  MEOX2  GATA5  MEF2A  MLLT3  YBX3  TFAP2B  ERCC6  VEZF1  TP53  RAD21  SEMA4D  B2M  NRP1  IL15RA  HLA-B  TNFRSF9  TREM1  ALB  SLC8A1  AQP1 | EDN1  EDN1  EDN1  HLA-DQA1  HLA-DQA1,HLA-DQB1  EDN1,HLA-DQA1,RAB27A  EDN1,HLA-DQA1,HLA-DQB1,RAB27A  EDN1  EDN1  MSI1  HLA-DQA1,HLA-DQB1  EDN1,HLA-DQA1  HLA-DQA1  OGG1  GPC4  HLA-DQA1,HLA-DQB1  EDN1  EDN1  EDN1  EDN1  EDN1  EDN1  OGG1  EDN1  EDN1  EDN1  EDN1  HSPA4L  EDN1  EDN1  EDN1  GPC4,HSPA4L  EDN1  EDN1,HLA-DQB1  HLA-DQB1  CACNA1I  CACNA1I  EDN1  HLA-DQA1  GPC4,HSPA4L  EDN1  EDN1  EDN1  HSPA4L  DBI,EDN1,HLA-DQA1,OGG1  EDN1,HLA-DQB1  DBI,EDN1,MSI1  DBI  EDN1  EDN1  EDN1  HLA-DQB1  EDN1  NAP1L3  HLA-DQA1  RAB27A  HLA-DQA1,HLA-DQB1  EDN1  HLA-DQA1,RAB27A  RAB27A  EDN1  EDN1  HLA-DQA1,HLA-DQB1  HSPA4L  HLA-DQA1,HLA-DQB1  HLA-DQA1,HLA-DQB1  RAB27A  HLA-DQA1  EDN1  GPC4  EDN1  EDN1  HSPA4L  EDN1  EDN1,HLA-DQA1,RAB27A  EDN1,HLA-DQA1  EDN1  HLA-DQA1,HLA-DQB1  DBI,EDN1  HLA-DQA1,HLA-DQB1  HLA-DQB1,OGG1  DBI  HLA-DQA1  EDN1  EDN1  HLA-DQB1,OGG1  HLA-DQA1,HLA-DQB1  HLA-DQB1  HLA-DQB1  EDN1  EDN1  EDN1  EDN1  HLA-DQB1  EDN1  OGG1  EDN1  DBI,EDN1,HLA-DQA1,HSPA4L,WSB2  HLA-DQA1,HLA-DQB1  HLA-DQB1  HLA-DQA1,HLA-DQB1  HLA-DQB1  EDN1  EDN1  HLA-DQB1  EDN1,MCF2L2  EDN1  EDN1  HSPA4L |
