# Supplementary material for: Clinical and transcriptional recovery profiles in pediatric and adult multiple sclerosis patients
Source: Ann Clin Transl Neurol. 2020 Nov 16;8(1):81–94. doi: 10.1002/acn3.51244 (PMC7818128; doi:10.1002/acn3.51244)
Supplement: Supplementary file 2 — Table S2. DEGs between pediatric and adult healthy controls 257 DEGs between healthy pediatric (age ≤ 18 years old) and adults (age 19–40 years old) controls. DEGs – Differentially Expressed Genes [file ACN3-8-81-s002.docx]

| **DEGs** | **Fold change direction**  **(pediatric/adult)** |
| --- | --- |
| ARL4C, BAT2D1, RPLP2, PPP3CC, EEF1D, MED13L, NUFIP1, MSL2, RPL14, RPL27A, C20orf11, CDC42SE1, JAG2, SLC9A8, TESK2, ANKRD12, LMBR1L, HNRNPA1, VPS13D, YLPM1, PGK1, MCL1, EIF5B, COL1A1, SUPV3L1, FUSIP1, NIPBL, BAG5, ERAP1, DENND5A, MEF2A, LOC100287697, CNOT2, RPAIN, HNRNPD, SOX4, MAGOH2, SRRM1, TTR, hCG_1730474, HNRNPH2, WTAP, JARID2, BMP8A /// BMP8B, PDPK1, RBM26, SKI, TNKS2, COX4I1, EZR, RBM39, **EDN1**, SFPQ, COL11A2, HNRNPL , USP4, IL24, NECAP1, ZMYM2, SENP3, SFRS6, PABPN1, CSNK1G2, NPIPL3, CDK2, DBF4, LRIG2, SFRS18, CLASP1, ZNF281, ILKAP, VPRBP, BNIP1, HSPA9, RHEB, ADAM12, COX5B, REL, ZBTB10, RPS11, PRDM12, TMEM97, FGFR1, TRAF4, BRD1, ADNP2, RAPGEF6, TBL1X, C6orf62, MTCH1, AP3D1, ATP6V0E1, RNF24, FBXW2, PPP3R1, CBLL1, IGHG1, GADD45B, C17orf63, EEF1E1, RASGRP2, HNRNPH3, PCIF1, FOXO1, C3orf37, JHDM1D, CDH16, TGIF1, CEBPD, TNFRSF4, CYLD, RAB11FIP2, PDCD4, SBNO1, MAFG, KDM3B, CLCN6, CDC5L, TERF2IP, GADD45G, PFKFB2, SFRS12, DCUN1D2, FAM53C, RNF103, DDX21, JOSD1, NUP153, DNAH3, ZNF3, MAPK8, IRGQ, PDZD3, EDEM1, HNRNPH1, TRA2A, KIAA1109, MAFF, CCK, MAP4K5, SRRT, MED6, MTMR10, C1orf68, DMBT1, GNB2L1, DNAJB9, RIN3, PRDM2, KIAA1609, ELL2, PFDN4, SLTM, EFNB3, SLC25A36 , EXOC7, DDIT3 /// NR1H3, ZC3H7A, NCAPH2, SEC31A, ATP10B, APOE, SERTAD2, DNMT3A, RTF1, C10orf110, ELF2, RNF38, FAM120C, ZZEF1, PTBP2, ProSAPiP1, FUBP1, PSMD11, SSBP1, RNF10, PTP4A1, PTS, RICS, S100A8, CDC2L5, EIF5, EAPP, GGA1, CREM, TRA2B, RBM25, ATXN2L  ATP5I, PSME3, FKBP1A, RAB2A, CRBN, KANK2, PNPLA2, PRKG2, PRKCSH, BBX, SERBP1, ROCK2, KDM2A, TGOLN2, ASXL2, C1orf69, TMSB10, ATP6V1A, NEURL, SPCS2, USP1, LOC100293596, ALDH6A1, IPO7 , MTF2, GNAS, IL32, PKM2, AP2B1, CLSTN1, HLA-F, OLA1, RAP2A, HP1BP3, GIMAP4, CX3CR1, SFRS2IP, GIMAP6, VAMP8, EIF3G, DLX2, PTPN11, EIF3A, C14orf101, PTPN12, EIF2S2, BANF1, CAPZB, CHPF, IL7R, UBL3, SPDEF, PSMD4, DYRK1B, SSH1, HOXC8, SCNN1A, CD74, GATAD1, EDEM3, COX6A2, DYNC1I2, ARL6IP5, MAP3K7, ST6GAL1, PEA15, HDLBP, YWHAE, DCAF15, PDIA6, URM1, LRP6, EPS8L1, CEP350, PPP2R3A, PPIA, PSMG2, ZMYND11, PTER, PTPRC, MORN1, ORMDL2, IL17RA, C2orf49, RC3H2, CAPN6, RAC2, ICAM2, TNFSF10, CLTA, GPR144, COPA, ADD3, CSF1R, ASH2L, DUX4, THTPA, ATP5H, UBE2L3, CORO1A, GIMAP5, AP1S2, NDUFB5, EIF4G2, SCYL2, GP1BB, GLRX, SYNCRIP, DDX18, AHCYL1, RHOQ, VEZF1 | UP  DOWN |

**Supplementary Table 2. DEGs between pediatric and adult healthy controls**
