# Supplementary material for: Clinical and transcriptional recovery profiles in pediatric and adult multiple sclerosis patients
Source: Ann Clin Transl Neurol. 2020 Nov 16;8(1):81–94. doi: 10.1002/acn3.51244 (PMC7818128; doi:10.1002/acn3.51244)
Supplement: Supplementary file 3 — Table S3. Upstream regulators of 28 DEGs associated with no recovery. Upstream regulators analysis revealed 152 potential regulators for 28 DEGs between POMS and AOMS patients that showed no clinical recovery after their first relapse. DEGs – Differentially Expressed Genes, POMS – Pediatric Onset Multiple Sclerosis, AOMS – Adult Onset Multiple Sclerosis [file ACN3-8-81-s003.docx]

**Supplementary Table 3. Upstream regulators of 28 DEGs associated with no recovery.**

| **Upstream regulators** | **Target molecules** |
| --- | --- |
| PAX5  CD40  PRDM1  IL4  FLT3LG  SOX11  IL7R  LPS  CD79A  ID2  LY9  POU2AF1  SPI1  EBF1  MSC  CR2  IL7  STAT6  BNIP3L  YY1  Igm  LYN  RAG1  TCF3  TNFRSF13B  rituximab  BCL6  ID3  SMARCC1  CDKN1B  MTA3  POU2F1  glucocorticoid  TADA2B  HHIP  Igha  EBF1-FOXO1-TCF3  1,2,3,6,7,8-HCDF  IL4R  IL5  bryostatin 1  SERPINA1  IRF4  BRD2  IGLL1/IGLL5  CD79B  Ly6a  TCF12  zymosan  Bay 11-7082  IL13  galactosylceramide  HLA-DR  UBP1  H3F3A  TWSG1  LAMTOR3  TNFSF13B  NFKB2  CD40LG  REL  LY75  SDHA  IGL  IGKC  sodium alum  2V3D  USF1  SCAP  PARP14  SAMSN1  PIGR  HOXC11  E. coli B4 LPS  PPARG  POU2F2  IL6  EHF  Tlr  QRFP  EN2  CTNNB1  bezafibrate  METRNL  ZNF423  PAX9  STAT5a/b  N-cor  Bcl9-Cbp  CD8A  PPP2R1A  dovitinib  APH1A  BCO2  CD22  FTH1  FURIN  D-sphingosine  salmonella minnesota R595 lipopolysaccharides  GH1  TNFRSF13C  UBD  zeaxanthin  bile salt  TICAM1  Sb202190  FGF1  Il8r  Adaptor protein 1  BCO1  brodalumab  TRAF5  HNRNPD  RFX5  SU5402  BCR (complex)  IL10  SP1  ASIP  SPINT1  1-NITC  IKZF1  ITGB1  IL2  Srebp  EN1  CD70  PIAS4  CG  AGPAT2  SATB2  KDM4A  MAPK8IP3  CFB  EHHADH  CNOT3  AXIN1  SDCBP  IL1RL1  PTK2B  batimastat  ADIPOQ  Immunoglobulin  TET1  CD81  LRP5  SATB1  AGN194204  CD200  PAX2  ADIPOR1  linalool  resiquimod  MED1  PKD1  TRA  MGAT5  GNL3  KAT2A  DEHP  KLF2  CHD7  FUT8  SOX2-OCT4-NANOG  BCOR  DRAP1  RNASE1  CD47  RNASEL  SEMA7A  Tcf7  linolenic acid  TP53 | BLNK,CD19,CD22,CD79A,CR2,FCER2  CD22,CR2,FCER2,IL4R,SAMSN1,TCL1A  CD19,CD22,CD79A,FCER2,IGHM,PAX5  ACSL1,CD79A,CR2,FCER2,FPR2,IL4R,MS4A1,PAWR,PAX5,SAMSN1  CD19,CD79A,PAX5,TCF4  BCL7A,CD19,MS4A1,PAX5  CD19,CD79A,IGHM,PAX5  ABCB4,ACSL1,CD22,CR2,FCER2,FPR2,IGHM,IL4R,PAX5,SAMSN1,STAP1,TCF4  CD79A,IGHM  CD19,CD79A,IL4R,PAWR,TCF4  CD19,CR2  CD79A,IGHM,PAX5  BCL7A,BLNK,CD19,CD79A,MS4A1  CD19,CD79A,IGHM,PAX5  CD19,CD79A,PAX5  CD19,CR2  BLNK,CD19,IGHM,PAX5  ACSL1,CR2,FCER2,IL4R,SAMSN1  BLNK,CD19,CD22  BLNK,CD22,CR2,PAWR,TCF4  CD22,PAX5,SAMSN1  CD19,CD22,CR2  CD79A,IGHM  BLNK,CD79A,CR2,IGHM,PAX5  CD19,IGHM  IGHM,MS4A1  BLNK,CD19,FCER2,IL4R  CD79A,IL4R,PAWR,TCF4  CD79A,PAX5  BLNK,PAX5,TCF4  BLNK,CD19  ACSL1,CD79A,CR2  CR2,FCER2,IGHM,TCL1A  CD19  PAX5  IGHM  PAX5  IGHM  ACSL1,FCER2  CD79A,IGHM,IL4R,PAX5  CD22,MS4A1  CR2,FCER2  FCER2,MS4A1,PAX5  CR2,PAWR  IGHM  IGHM  IGHM  FCER2  APH1B,BLNK,IGHM,PAX5  BLNK,PAX5  FPR2,IL4R  FPR2,SAMSN1  ACSL1,FCER2,FPR2,SAMSN1  PAX5  IGHM  CD79A  CD79A  PAX5  TCL1A  CR2,FCER2  CR2,FCER2  FCER2,IL4R,PAWR,SAMSN1  CR2,FCER2,IGHM  FCER2  CD19  IL4R  IL4R  IL4R  PAWR  ABCB4,ACSL1,CR2,FPR2,IGHM,IL4R,MS4A1,PCDH9,SAMSN1  CR2,MS4A1  ACSL1,IGHM  FCER2  FCER2,TCF4  CD79A  PAWR  ABCB4,FPR2,SAMSN1  ACSL1,APH1B,CR2,TCF4  CD22,MS4A1  CD79A,FCER2,FPR2,IGHM,IL4R  BLNK,TCF4  FPR2,PAX5  ACSL1  PAX5  HTATIP2,IGHM,PCDH9,TCF4,TCL1A  ABCB4,ACSL1  ACSL1  CD79A  CD19  IL4R,PAX5  ACSL1,CR2  TCF4  IGHM  FPR2  CR2  APH1B  HTATIP2  CR2  ABCB4  IL4R  IL4R  ACSL1,FPR2  CD19,IL4R  CR2  ACSL1  HTATIP2  ABCB4  ACSL1,FPR2  FCER2,FPR2  CR2,TCF4  CR2  CR2  HTATIP2  IL4R  FCER2  CR2  CR2  CR2  FCER2,TCL1A  FCER2,HTATIP2,IL4R  ACSL1,CR2,FPR2,IL4R  TCF4  TCF4  ABCB4  BLNK,PAX5  IGHM,TCF4  ABCB4,FPR2,IL4R,PAX5  ACSL1  PAX5  CR2  PCDH9  ACSL1,IL4R,MARCH3  ACSL1  IGHM  TCL1A  PAX5  CR2  ACSL1  PAX5  TCF4  TCF4  PAWR  IGHM  FCER2  ACSL1,IL4R  ACSL1,IL4R,PAX5  TCL1A  CD19  TCF4  BLNK,IL4R  IGHM,IL4R  IL4R  PAX5  IL4R  ACSL1  FCER2,STAP1  BCL7A,CD79A  ABCB4,PCDH9  CD79A  IL4R  TCL1A  CD19  ACSL1,TCF4  CR2,TCF4  APH1B  IGHM  TCL1A  MS4A1  BLNK  FCER2  FCER2  PCDH9  IL4R  TCL1A  ACSL1  ABCB4,ACSL1,IL4R,PAWR,SQOR,TCF4,TCL1A |
